# Supplementary material for: Human foraging strategies flexibly adapt to resource distribution and time constraints
Source: Cogn Affect Behav Neurosci. 2025 Sep 22;26(1):43–63. doi: 10.3758/s13415-025-01347-4 (PMC12847125; doi:10.3758/s13415-025-01347-4)
Supplement: Supplementary file 1 — (pdf 942 KB) [file 13415_2025_1347_MOESM1_ESM.pdf]

Supplementary materials

Human foraging strategies flexibly adapt to resource distribution and time constraints

Valeria Simonelli*[a,b], Davide Nuzzi*[a], Gian Luca Lancia[a], Giovanni Pezzulo†[a]

[a] Institute of Cognitive Sciences and Technologies, National Research Council, Rome, Italy
[b] University of Rome "La Sapienza", Rome, Italy

*These authors contributed equally to this work
†Corresponding author: giovanni.pezzulo@istc.cnr.it

A Supplementary analyses

A.1 Supplementary tables of the analysis reported in Section 3.1

Here, we report the estimated parameters and statistics of linear mixed-effects modeling on six dependent variables: total coins collected, boxes collected in the first area, total boxes collected, average coins per box, number of visited areas, and average box collection time. This analysis considers the effects of our manipulations, namely the battery length, either long or short, and environmental richness, either rich or mixed.

	Coeff.	SE	t	2.5_ci	97.5_ci	P-val
Intercept	34.703	1.205	28.791	32.340	37.065	< 0.001
LongBattery	28.356	0.673	42.161	27.038	29.674	$\mathbf{< 0.001}$
RichEnvironment	7.241	0.673	10.767	5.923	8.559	$\mathbf{< 0.001}$
LongBattery × RichEnvironment	5.600	0.951	5.888	3.736	7.464	$\mathbf{< 0.001}$

Table S1: Estimated parameters and statistics of linear mixed-effects modeling on the total number of coins gained per trial.

	Coeff.	SE	t	2.5_ci	97.5_ci	P-val
Intercept	2.238	0.115	19.380	2.012	2.465	< 0.001
LongBattery	0.600	0.081	7.431	0.442	0.758	$\mathbf{< 0.001}$
RichEnvironment	0.318	0.081	3.934	0.159	0.476	$\mathbf{< 0.001}$
LongBattery × RichEnvironment	0.194	0.114	1.700	-0.030	0.418	0.089

Table S2: Estimated parameters and statistics of linear mixed-effects modeling on the number of boxes collected in the first area.

	Coeff.	SE	t	2.5_ci	97.5_ci	P-val
Intercept	5.582	0.206	27.057	5.178	5.987	< 0.001
LongBattery	5.432	0.113	48.045	5.211	5.654	$\mathbf{< 0.001}$
RichEnvironment	0.244	0.113	2.159	0.023	0.466	**0.031**
LongBattery × RichEnvironment	-0.018	0.160	-0.110	-0.331	0.296	0.912

Table S3: Estimated parameters and statistics of linear mixed-effects modeling on the number of boxes opened per trial.

	Coeff.	SE	t	2.5_ci	97.5_ci	P-val
Intercept	6.231	0.044	143.214	6.146	6.316	< 0.001
LongBattery	-0.454	0.032	-14.167	-0.391	0.851	$\mathbf{< 0.001}$
RichEnvironment	0.999	0.032	31.182	0.936	1.061	$\mathbf{< 0.001}$
LongBattery × RichEnvironment	0.002	0.045	0.042	-0.087	0.091	0.966

Table S4: Estimated parameters and statistics of linear mixed-effects modeling on the average coins collected per box.

	Coeff.	SE	t	2.5_ci	97.5_ci	P-val
Intercept	2.965	0.081	36.730	2.807	3.123	< 0.001
LongBattery	0.765	0.044	17.342	0.678	0.851	**< 0.001**
RichEnvironment	-0.050	0.044	-1.134	-0.136	0.036	0.257
LongBattery × RichEnvironment	-0.015	0.062	-0.236	-0.137	0.108	0.814

Table S5: Estimated parameters and statistics of linear mixed-effects modeling on the number of visited areas.

	Coeff.	SE	t	2.5_ci	97.5_ci	P-val
Intercept	3.324	0.109	30.413	3.110	3.539	< 0.001
LongBattery	0.263	0.073	3.597	0.120	0.406	**< 0.001**
RichEnvironment	-0.022	0.073	-0.300	-0.165	0.121	0.764
LongBattery × RichEnvironment	-0.014	0.103	-0.136	-0.216	0.188	0.892

Table S6: Estimated parameters and statistics of linear mixed-effects modeling on the average box collection time.

Environment	Battery	Contrast	Estimate	Std Error	p-value
Mixed	.	Short - Long	-28.356	0.673	**< 0.001**
Rich	.	Short - Long	-33.956	0.673	**< 0.001**
.	Short	Mixed - Rich	-7.241	0.673	**< 0.001**
.	Long	Mixed - Rich	-12.841	0.673	**< 0.001**

Table S7: Pairwise post-hoc comparisons for the variable: total coins

Environment	Battery	Contrast	Estimate	Std Error	p-value
Mixed	.	Short - Long	-0.600	0.081	**< 0.001**
Rich	.	Short - Long	-0.794	0.081	**< 0.001**
.	Short	Mixed - Rich	-0.318	0.081	**< 0.001**
.	Long	Mixed - Rich	-0.512	0.081	**< 0.001**

Table S8: Pairwise post-hoc comparisons for the variable: boxes collected in first area

Environment	Battery	Contrast	Estimate	Std Error	p-value
Mixed	.	Short - Long	-5.432	0.113	**< 0.001**
Rich	.	Short - Long	-5.415	0.113	**< 0.001**
.	Short	Mixed - Rich	-0.244	0.113	0.118
.	Long	Mixed - Rich	-0.226	0.113	0.170

Table S9: Pairwise post-hoc comparisons for the variable: boxes collected

Environment	Battery	Contrast	Estimate	Std Error	p-value
Mixed	.	Short - Long	0.454	0.032	**< 0.001**
Rich	.	Short - Long	0.452	0.032	**< 0.001**
.	Short	Mixed - Rich	-0.999	0.032	**< 0.001**
.	Long	Mixed - Rich	-1.001	0.032	**< 0.001**

Table S10: Pairwise post-hoc comparisons for the variable: average coins per box

Environment	Battery	Contrast	Estimate	Std Error	p-value
Mixed	.	Short - Long	-0.765	0.044	**$<$ 0.001**
Rich	.	Short - Long	-0.750	0.044	**$<$ 0.001**
.	Short	Mixed - Rich	0.050	0.044	0.695
.	Long	Mixed - Rich	0.065	0.044	0.459

Table S11: Pairwise post-hoc comparisons for the variable: number of visited areas

Environment	Battery	Contrast	Estimate	Std Error	p-value
Mixed	.	Short - Long	-0.263	0.073	0.001
Rich	.	Short - Long	-0.249	0.073	0.003
.	Short	Mixed - Rich	0.022	0.073	0.997
.	Long	Mixed - Rich	0.036	0.073	0.980

Table S12: Pairwise post-hoc comparisons for the variable: time between boxes

A.2 Supplementary tables of the analysis reported in Section 3.2

In this section, we present the estimated parameters and statistics of linear mixed-effects modeling on six dependent variables: total coins collected, boxes collected in the first area, total boxes collected, average coins per box, number of visited areas, and average box collection time. The analysis considers the effect of our manipulations taking into account the richness of the first area visited by participants, in mixed environment trials.

	Coeff.	SE	t	2.5_ci	97.5_ci	P-val
Intercept	333.732	1.122	30.063	31.533	35.931	< 0.001
LongBattery	29.008	0.836	34.716	27.370	30.646	**$<$ 0.001**
FirstAreaRich	1.931	0.822	2.349	0.320	3.542	**0.019**
LongBattery × FirstAreaRich	-1.345	1.159	-1.161	-3.616	0.926	0.246

Table S13: Estimated parameters and statistics of linear mixed-effects modeling on the total number of coins gained per trial.

	Coeff.	SE	t	2.5_ci	97.5_ci	P-val
Intercept	1.632	0.115	14.241	1.408	1.857	< 0.001
LongBattery	0.290	0.100	2.893	0.093	0.486	**0.004**
FirstAreaRich	1.205	0.098	12.239	1.012	1.398	**$<$ 0.001**
LongBattery × FirstAreaRich	0.479	0.139	3.451	0.207	0.751	**0.001**

Table S14: Estimated parameters and statistics of linear mixed-effects modeling on the number of boxes collected in the first area.

	Coeff.	SE	t	2.5_ci	97.5_ci	P-val
Intercept	5.606	0.217	25.779	5.180	6.032	< 0.001
LongBattery	5.404	0.159	33.997	5.092	5.715	**$<$ 0.001**
FirstAreaRich	-0.047	0.156	-0.301	-0.354	0.259	0.763
LongBattery × FirstAreaRich	0.056	0.220	0.255	-0.376	0.488	0.799

Table S15: Estimated parameters and statistics of linear mixed-effects modeling on the number of boxes opened per trial.

	Coeff.	SE	t	2.5_ci	97.5_ci	P-val
Intercept	6.002	0.053	112.410	5.898	6.107	< 0.001
LongBattery	-0.263	0.053	-4.953	-0.367	-0.159	**$<$ 0.001**
FirstAreaRich	0.455	0.052	8.724	0.353	0.557	**$<$ 0.001**
LongBattery × FirstAreaRich	-0.385	-0.241	-5.242	-0.530	-0.241	**$<$ 0.000**

Table S16: Estimated parameters and statistics of linear mixed-effects modeling on the average coins collected per box.

	Coeff.	SE	t	2.5_ci	97.5_ci	P-val
Intercept	3.129	0.082	38.353	2.969	3.289	< 0.001
LongBattery	0.718	0.064	11.199	0.592	0.844	**< 0.001**
FirstAreaRich	-0.326	0.063	-5.171	-0.450	-0.202	**< 0.001**
LongBattery × FirstAreaRich	0.111	0.089	1.245	-0.064	0.285	0.214

Table S17: Estimated parameters and statistics of linear mixed-effects modeling on the number of visited areas.

	Coeff.	SE	t	2.5_ci	97.5_ci	P-val
Intercept	3.223	0.119	27.023	2.989	3.456	< 0.001
LongBattery	0.291	0.109	2.679	0.078	0.505	**0.008**
FirstAreaRich	0.202	0.107	1.889	-0.008	0.412	0.059
LongBattery × FirstAreaRich	-0.068	0.151	-0.452	-0.364	0.228	0.652

Table S18: Estimated parameters and statistics of linear mixed-effects modeling on the average box collection time.

First Area	Battery	Contrast	Estimate	Std Error	p-value
Poor	.	Short - Long	-29.008	0.836	**< 0.001**
Rich	.	Short - Long	-27.663	0.797	**< 0.001**
.	Short	Poor - Rich	-1.931	0.822	0.074
.	Long	Poor - Rich	-0.586	0.825	0.926

Table S19: Pairwise post-hoc comparisons for the variable: total coins

First Area	Battery	Contrast	Estimate	Std Error	p-value
Poor	.	Short - Long	-0.290	0.100	0.016
Rich	.	Short - Long	-0.769	0.095	**< 0.001**
.	Short	Poor - Rich	-1.205	0.098	**< 0.001**
.	Long	Poor - Rich	-1.684	0.099	**< 0.001**

Table S20: Pairwise post-hoc comparisons for the variable: boxes collected in first area

First Area	Battery	Contrast	Estimate	Std Error	p-value
Poor	.	Short - Long	-5.404	0.159	**< 0.001**
Rich	.	Short - Long	-5.460	0.152	**< 0.001**
.	Short	Poor - Rich	0.047	0.156	0.997
.	Long	Poor - Rich	-0.009	0.157	1.000

Table S21: Pairwise post-hoc comparisons for the variable: boxes collected

First Area	Battery	Contrast	Estimate	Std Error	p-value
Poor	.	Short - Long	0.263	0.053	**< 0.001**
Rich	.	Short - Long	0.648	0.051	**< 0.001**
.	Short	Poor - Rich	-0.455	0.052	**< 0.001**
.	Long	Poor - Rich	-0.069	0.052	0.560

Table S22: Pairwise post-hoc comparisons for the variable: average coins per box

First Area	Battery	Contrast	Estimate	Std Error	p-value
Poor	.	Short - Long	-0.718	0.064	$<$ **0.001**
Rich	.	Short - Long	-0.829	0.061	$<$ **0.001**
.	Short	Poor - Rich	0.326	0.063	$<$ **0.001**
.	Long	Poor - Rich	0.215	0.063	0.003

Table S23: Pairwise post-hoc comparisons for the variable: number of visited areas

First Area	Battery	Contrast	Estimate	Std Error	p-value
Poor	.	Short - Long	-0.291	0.109	0.030
Rich	.	Short - Long	-0.223	0.104	0.121
.	Short	Poor - Rich	-0.202	0.107	0.217
.	Long	Poor - Rich	-0.134	0.107	0.615

Table S24: Pairwise post-hoc comparisons for the variable: time between boxes

A.3 Supplementary analysis, restricted to trials in which the first visited area was rich, in both rich and mixed environments

In this section, we present the estimated parameters and statistics of linear mixed-effects modeling on boxes collected in the first area. The analysis considers the effect of our manipulations, namely the battery length, either long or short, and environmental richness, either rich or mixed, but is restricted to cases in which the first visited area was rich (which is always true in the rich environment, but only approximately half of the times in the mixed environment). See also Figure S2.

	Coeff.	SE	t	2.5_ci	97.5_ci	P-val
Intercept	2.842	0.131	21.621	2.585	3.100	< 0.001
LongBattery	0.766	0.097	7.884	0.576	0.957	**< 0.001**
RichEnvironment	-0.286	0.086	-3.331	-0.455	-0.118	**0.001**
LongBattery × RichEnvironment	0.038	0.120	0.315	-0.197	0.273	0.753

Table S25: Estimated parameters and statistics of linear mixed-effects modeling on the number of boxes collected in the first area.

A.4 Supplementary tables of the analysis reported in Section 3.4

Fixed Effect	Coeff	Odds Ratio	[0.025	0.975]	z-stat	p-value
(Intercept)	0.027	1.027	0.677	1.559	0.127	0.899
Uncertainty Resolved	0.546	1.726	1.097	2.716	2.362	**0.018**
Rich Environment	0.984	2.674	1.978	3.614	6.396	**< 0.001**
Current Area Rich	-2.210	0.110	0.074	0.162	-11.036	**< 0.001**
Battery Long	-0.661	0.516	0.370	0.720	-3.889	**< 0.001**
Box Number Current Area	1.173	3.231	2.692	3.879	12.579	**< 0.001**
Uncertainty Resolved × Current Area Rich	-0.993	0.371	0.207	0.664	-3.339	**0.001**
Uncertainty Resolved × Battery Long	-0.655	0.519	0.346	0.779	-3.164	**0.002**
Uncertainty Resolved × Box Number Current Area	0.352	1.421	1.146	1.762	3.206	**0.001**
Group Variance	0.668					

Table S26: Estimated parameters and statistics of a generalized linear mixed-effects model predicting the probability of switching area after box opening when in the second area.

Rich Environment	Current Area Rich	Uncertainty Resolved	Contrast	Estimate	Std	p-value
.	Rich	Resolved	Rich - Mixed	-0.98	0.15	**< 0.001**
Mixed	Rich	.	Resolved - Unresolved	0.41	0.19	**0.030**
Mixed	Poor	.	Resolved - Unresolved	-0.58	0.21	**0.005**
Mixed	.	Resolved	Rich - Poor	3.20	0.23	**< 0.001**
Mixed	.	Unresolved	Rich - Poor	2.21	0.20	**< 0.001**

Table S27: Pairwise post-hoc comparisons between levels of Rich Environment, Current Area Rich, and Uncertainty Resolved from the generalized linear mixed-effects model on area-switching behavior.

Fixed Effect	Coeff	Odds Ratio	[0.025	0.975]	z-stat	p-value
(Intercept)	0.027	1.027	0.735	1.435	0.157	0.875
Uncertainty Resolved	1.979	7.232	4.356	12.008	7.648	**< 0.001**
Rich Environment	0.742	2.101	1.790	2.465	9.092	**< 0.001**
Current Area Rich	-2.433	0.088	0.067	0.115	-17.545	**< 0.001**
Battery Long	-0.747	0.474	0.390	0.576	-7.527	**< 0.001**
Box Number Current Area	1.285	3.616	3.245	4.029	23.302	**< 0.001**
Area number	-0.024	0.976	0.720	1.324	-0.156	0.876
Uncertainty Resolved × Battery Long	-0.349	0.705	0.559	0.890	-2.943	**0.003**
Uncertainty Resolved × Box Number Current Area	0.005	1.005	0.891	1.133	0.081	0.935
Uncertainty Resolved × Current Area Rich	-1.807	0.164	0.093	0.291	-6.200	**< 0.001**
Uncertainty Resolved × Area Number	-1.241	0.289	0.198	0.422	-6.449	**< 0.001**
Area Rich × Area Number	0.079	1.082	0.723	1.620	0.384	0.701
Uncertainty Resolved × Area Rich × Area Number	0.917	2.502	1.573	3.981	3.871	**< 0.001**
Group Variance	0.600					

Table S28: Estimated parameters and statistics of a generalized linear mixed-effects model predicting the probability of switching area after box opening for all areas.

A.5 Supplementary Figures

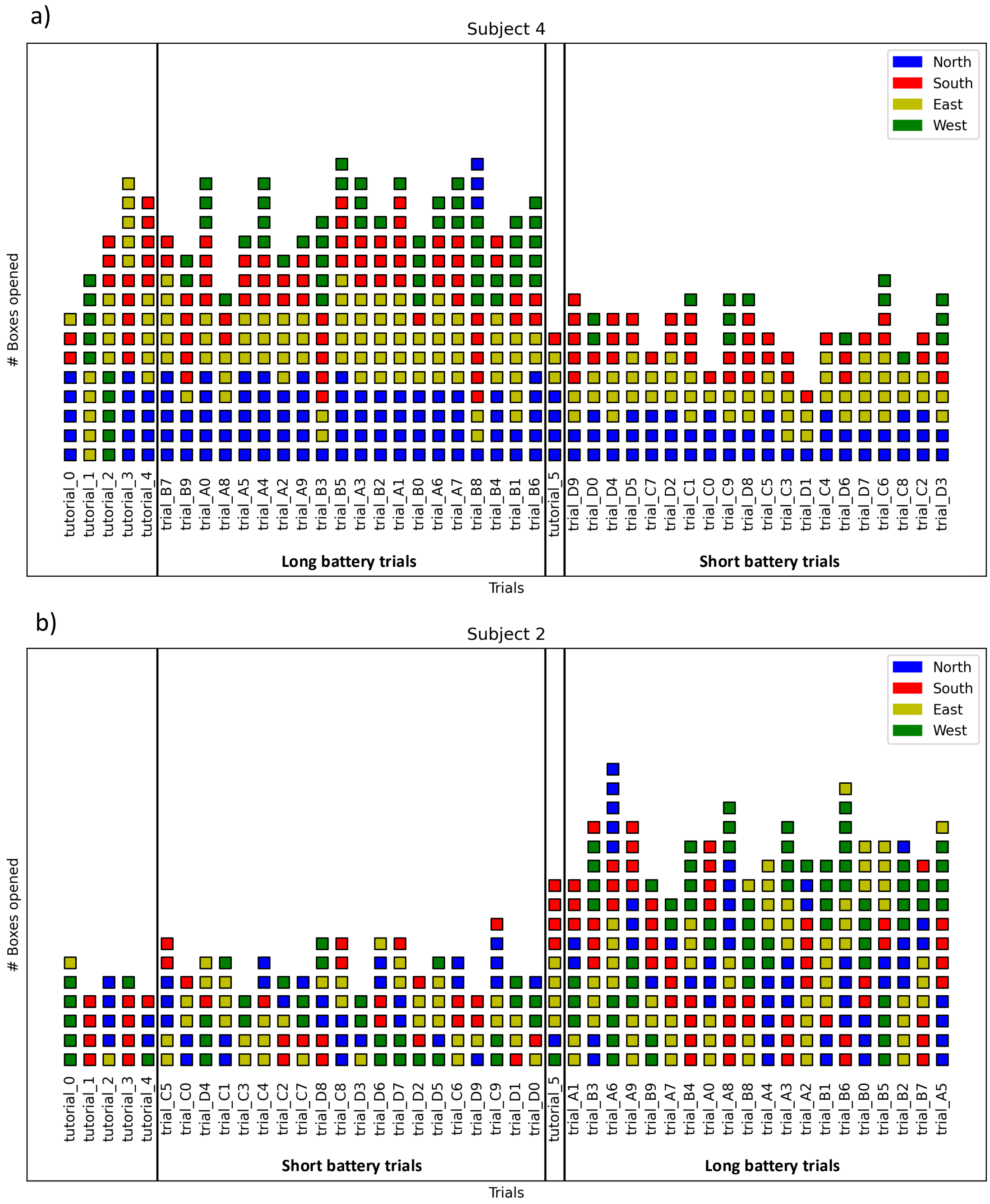

Figure S1: Box-opening patterns throughout the experiment for two representative participants employing different navigational strategies. Each square represents a box opened, with colors indicating the area to which the box belonged. (a) Subject 4 follows a stable, clockwise navigational pattern, while (b) Subject 2 exhibits an unstable navigational pattern, highlighting differences in area preferences and exploration strategies over time. See the main text for explanation.

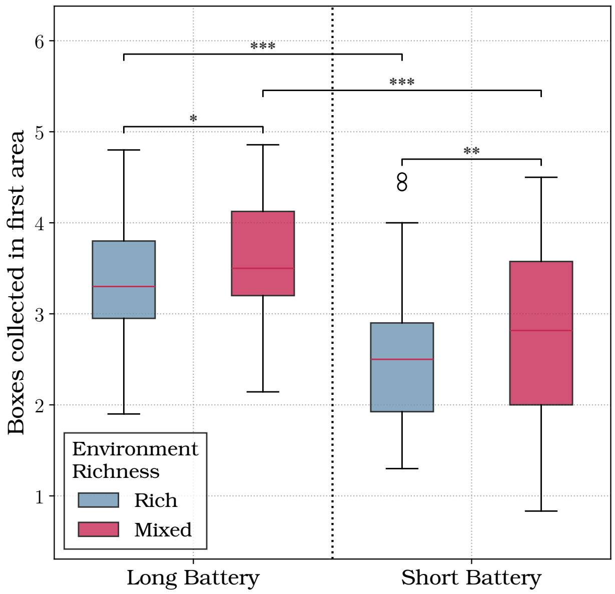

Figure S2: Boxes collected in the first area, only when it is rich, for both rich and mixed environments. See the main text for explanation.

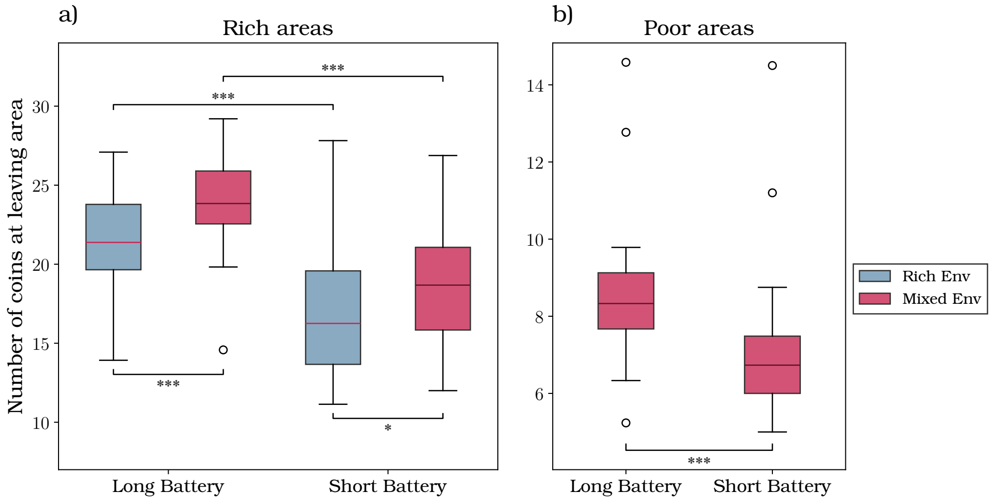

Figure S3: Number of coins collected by participants before leaving (a) rich or (b) poor areas. The number of collected coins changes significantly across conditions ($p = 0.041$ for the environment richness comparison in the short battery condition, $p < 0.001$ in all the other cases, Mann-Whitney U-test, FDR correction), suggesting that participants do not adopt a threshold-driven strategy to decide when to leave areas. See the main text for explanation.

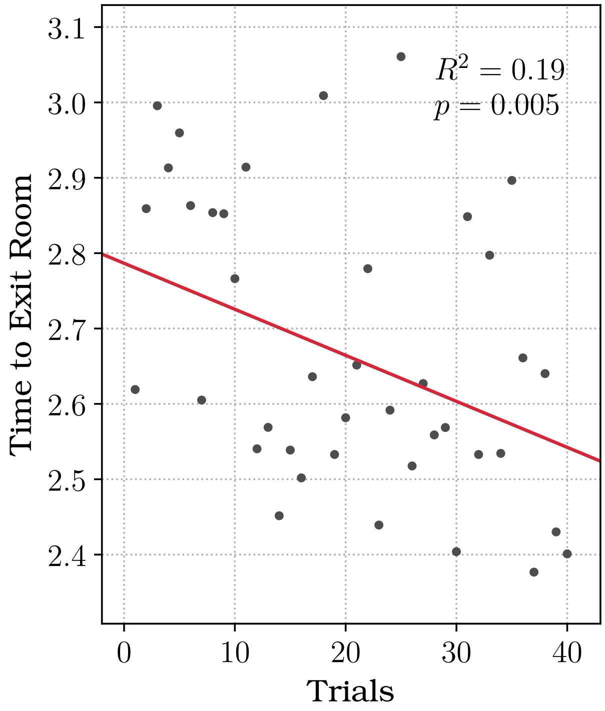

Figure S4: Participants' *exit time*: average time to leave a room after collecting the last treasure box within it, across different experimental conditions. The red line represents the linear regression fit, highlighting a significant decrease in time to exit as participants progressed through the trials ($R^2 = 0.19$, $p = 0.005$). See the main text for explanation.

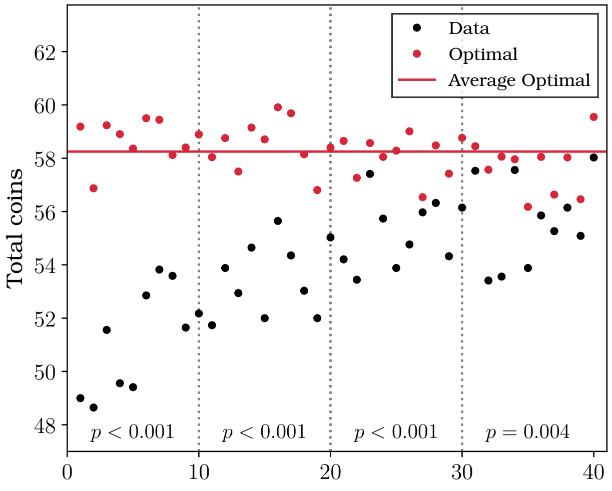

Figure S5: Participants' performance increases over time, approximating but not reaching the optimal agent. The plot compares the total coins earned by the optimal agent (red line is the average) and participants (black dots). Note that participants' data are the same as Figure 5.a. The results of four t-tests (shown at the bottom of the figure) show that the performance of the optimal agent is significantly better, across all the four periods considered. See the main text for explanation.
